# Image Mosaicking Approach for a Double-Camera System in the GaoFen2 Optical Remote Sensing Satellite Based on the Big Virtual Camera

**DOI:** 10.3390/s17061441

**Published:** 2017-06-20

**Authors:** Yufeng Cheng, Shuying Jin, Mi Wang, Ying Zhu, Zhipeng Dong

**Affiliations:** State Key Laboratory of Information Engineering in Surveying, Mapping and Remote Sensing, Wuhan University, Wuhan 430079, China; cyf_whu@126.com (Y.C.); yzhu1003@whu.edu.cn (Y.Z.); zhipengdong@foxmail.com (Z.D.)

**Keywords:** image mosaicking, double-camera system, big virtual camera, GaoFen2

## Abstract

The linear array push broom imaging mode is widely used for high resolution optical satellites (HROS). Using double-cameras attached by a high-rigidity support along with push broom imaging is one method to enlarge the field of view while ensuring high resolution. High accuracy image mosaicking is the key factor of the geometrical quality of complete stitched satellite imagery. This paper proposes a high accuracy image mosaicking approach based on the big virtual camera (BVC) in the double-camera system on the GaoFen2 optical remote sensing satellite (GF2). A big virtual camera can be built according to the rigorous imaging model of a single camera; then, each single image strip obtained by each TDI-CCD detector can be re-projected to the virtual detector of the big virtual camera coordinate system using forward-projection and backward-projection to obtain the corresponding single virtual image. After an on-orbit calibration and relative orientation, the complete final virtual image can be obtained by stitching the single virtual images together based on their coordinate information on the big virtual detector image plane. The paper subtly uses the concept of the big virtual camera to obtain a stitched image and the corresponding high accuracy rational function model (RFM) for concurrent post processing. Experiments verified that the proposed method can achieve seamless mosaicking while maintaining the geometric accuracy.

## 1. Introduction

Linear array push broom is a widely used imaging mode for the high resolution optical satellite (HROS). To increase the resolution, the camera is typically designed with a long focal length to achieve a narrow field angle. To increase the field of view (FOV), it is necessary to assemble additional TDI-CCD detectors together because the existing TDI-CCD detector cannot achieve the large field of view requirement, such as IKONOS, Pleiades-HR, Worldview series, ZiYuan3, etc. [1,2,3]. Another method to enlarge the field of view is to use double-cameras attached with a high-rigidity support concurrently with push broom imaging. Launched in 2014, the GaoFen2 (GF2) remote sensing satellite is a Chinese high resolution optical satellite equipped with a double-camera push broom imaging system resulting in a high resolution of 0.81 m; a 45 km width strip can be obtained, which is four times the size that IKNONS can obtain. However, the image of each individual camera in the double-camera system has an independent optical imaging system and imaging model, which creates additional work in the subsequent processing. Therefore, it is necessary to investigate the corresponding image mosaicking approach for the double-camera system.

Researchers have focused on investigations involving stitching several satellite images together [4,5]; there are two methods that are traditionally used. The first method is to rectify all the images to the same geodetic coordinate system, and then stitch the rectified images by image registration and mosaicking to produce the complete image. This method has high geometric accuracy but high complexity in image rectification and registration [6,7,8]. The second method is to utilize the overlapping regions to match the homonymy points for stitching the image while disregarding the geometric imaging model for each image [9,10,11]. The stitched image that results from this method lacks a rigorous imaging model, therefore, it has a poor geometric accuracy and cannot adequately meet the surveying and mapping requirements. Researchers have proposed an inner FOV stitching method based on the virtual detector line built in the camera coordinate system for multi-TDI-CCD mosaicking and achieved an ideal result, which provided the basic idea for exploring the mosaicking approach using multiple cameras [12,13]. The difference and difficulty for a double-camera system mosaicking compared with the multi-TDI-CCD mosaicking is the unstable relative installation relationship caused by the changing thermal environment, that is to say, even though we calibrated the relative installation relationship by one image scene, for other scenes, the relative installation relationship will probably has a tiny change, which will directly influent the mosaicking accuracy.

In this paper, we propose an image mosaicking approach for double-camera systems on the GF2 satellite based on the big virtual camera (BVC) in the satellite body coordinate system, which is built with no internal distortion and its FOV covers all the camera’s FOV. According to the original rigorous imaging model and the updated rational function model (RFM) by relative orientation of each camera, images obtained at the same time by the double-camera system are re-projected to the same big virtual camera coordinate system to produce the entire central projection stitched image by feather processing alone. Moreover, the high accuracy rational function model (RFM) of the stitched image can be obtained concurrently, which will provide the geometric information required for post processing. The proposed approach has been validated for precision and robustness, and it works under varying topographical conditions and achieves the goal of fully automated image data pretreatment on the ground.

## 2. Methodology

### 2.1. Rigorous Imaging Model for the Single Camera

**Figure 1:**
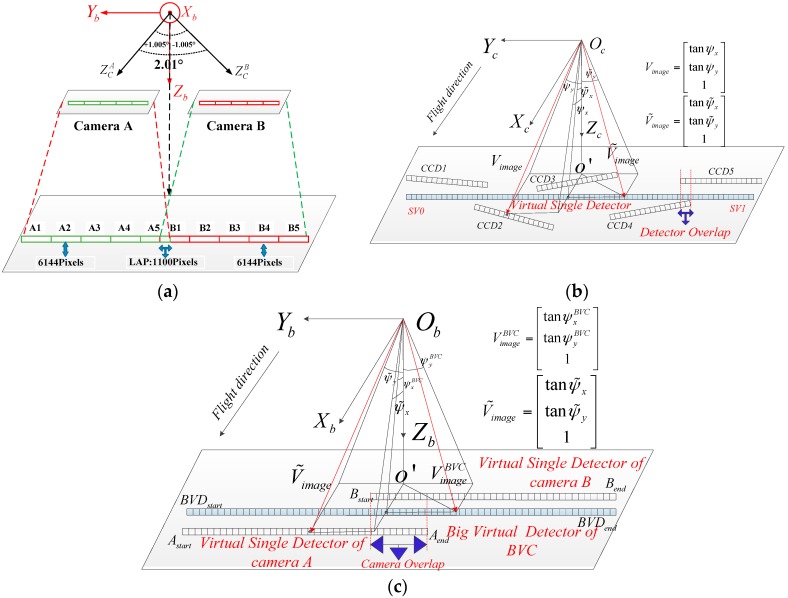
(**a**) The installation structure and configuration of the GF2 double-camera imaging system; (**b**) Schematic diagram of the virtual single detector in the single camera; (**c**) Schematic diagram of the big virtual detector in the big virtual camera.

In the double-camera push-broom imaging system on the GF2 satellite, the principal optic axes of the cameras are installed in the same plane as the greatest extent and the angle between them is 2.01°, as shown in Figure 1a. Therefore, double-cameras share the same orbit and attitude data when taking concurrent images, while each single camera has its own installation angles and internal optical parameters. The detailed information of the single camera is listed in Table 1.

The focal plane of the single camera is composed of five-collinear TDI-CCD detectors within the field of view and there are many overlapping pixels between the adjacent TDI-CCD detectors for mosaicking. The overlapping regions of the double-camera imaging system can be divided into the camera overlapping region and the detector overlapping region as shown in Figure 1b,c. The camera overlapping region is between the double cameras, and the detector overlapping region is between the two adjacent single TDI-CCD detectors in the same single camera. The virtual single detector (VSD) is used for the five-collinear TDI-CCD detectors to generate the entire stitched image from the single camera, while the big virtual camera (BVC) is used for the double-cameras to generate the entire stitched image from the double-camera imaging system. The goal of image mosaicking for the double-camera system is to seamlessly stitch ten image strips obtained simultaneously by the ten TDI-CCD detectors and to produce a complete wide coverage stitched image based on the virtual single detector and the big virtual camera.

The imaging parameters of the rigorous geometric imaging model can be divided into the satellite auxiliary data and the camera parameters. The satellite auxiliary data of time, attitude and orbit help to convert the satellite body coordinates into object coordinates, which determines the ray of light from the projection center to the ground points in the satellite body coordinate system. The interior LOS parameters help to convert the image coordinates into the camera coordinates, which determines the accurate LOS of each detector in the camera coordinate system [14,15]. For the single push-broom camera, the rigorous imaging model of each TDI-CCD detector can be determined as follows:

(1) $${\left[\begin{array}{c} X\\ Y\\ Z \end{array}\right]}_{WGS84}={\left[\begin{array}{c} X_{s}\\ Y_{s}\\ Z_{s} \end{array}\right]}_{WGS84}+\lambda R_{J2000}^{WGS84}R_{body}^{J2000}R_{camera}^{body}\left[\begin{array}{c} \tan\psi_{x}\\ \tan\psi_{y}\\ 1 \end{array}\right]$$

$$\left\{ \begin{array}{l} \tan\psi_{x}=a_{0}+a_{1}s+a_{2}s^{2}+a_{3}s^{3}\\ \tan\psi_{y}=b_{0}+b_{1}s+b_{2}s^{2}+b_{3}s^{3} \end{array}\right.$$

where $\psi_{x}$ and $\psi_{y}$ are the look angles [16,17] between the line of sight (LOS) and the vertical axis and horizontal axis of the focal plane. S is the sequence number of the single CCD detector, and $a_{0},a_{1},a_{2},a_{3}$ and $b_{0},b_{1},b_{2},b_{3}$ are the corresponding internal parameters. ${\left[\begin{array}{ccc} X & Y & Z \end{array}\right]}_{WGS84}^{T}$ is the object space WGS84 coordinate of the ground point. ${\left[\begin{array}{ccc} X_{S}(t) & Y_{S}(t) & Z_{S}(t) \end{array}\right]}^{T}$ is the position vector of the projection center in the WGS84 coordinate system, which is interpolated from ephemeris time observations. $R_{camera}^{body}$ is the installation matrix from the camera coordinate system to the satellite body coordinate system; and $R_{body}^{J2000}$ is the attitude matrix from the satellite body coordinate system to the J2000 coordinate system, which is interpolated from the attitude time observations under the J2000 coordinate system; and $R_{J2000}^{WGS84}$ represents the transformation matrix from the ECF coordinate system to the ECI coordinate system, which is based on the IAU 2000 precession-nutation model from the International Earth Rotation and Reference Systems Service (IERS) conventions.

The interior LOS of the virtual single detector can be easily determined by:

(2) $$\left\{ \begin{array}{l} \tan\tilde{\psi_{x}}=A_{0}\\ \tan\tilde{\psi_{y}}=B_{0}+B_{1}s \end{array}\right.$$

where $A_{0}$ is the mean value of the internal parameters ${\left(a_{0}\right)}_{i}$ i=1,2,3,4,5 of the five TDI-CCD detectors, by which the virtual single detector is placed in the center line of the multi-TDI-CCD along the flight direction. $B_{0}$ is equal to $b_{0}$ and $B_{1}$ is equal to $b_{1}$ of the leftmost TDI-CCD1, which eliminates internal distortion for the virtual single detector. Additionally, the original auxiliary data should undergo steady-state processing [18,19] to generate the virtual single images. The steady-state processing is a method to correct the attitude oscillation so as to increase RFM fitting precision for the rigorous imaging model.

### 2.2. Rigorous Imaging Model of the Big Virtual Camera

The single camera auxiliary data can also be applied to the BVC using steady-state processing. The BVC parameters consist of the exterior installation matrix and the interior LOS parameters. Based on the symmetrical installation relationship in the double-camera system shown in Figure 1a, we can assume that the BVC focal length equates to the single camera and the BVC field of view covers the entire imaging system, which is equivalent to placing a big virtual detector on the focal plane, as shown in Figure 1c. The exterior BVC installation matrix is considered the unit matrix, which means the LOS of each detector in the camera coordinate system is equal to the LOS in the satellite body coordinate system. To determine the interior BVC LOS, the number of virtual detectors and the field of view should be determined first. $N_{A}$ and $N_{B}$ represent the number of detectors of the virtual single detectors A and B. According to the design criterion, the number of detectors in the camera overlapping region in the satellite body coordinate system is $N_{Camera}$. Therefore, the number of BVC detectors is:

(3) $$N_{V}=N_{A}+N_{B}-N_{Camera}$$

Based on the above analysis, the LOS ${\left[\begin{array}{ccc} x_{i}(s) & y_{i}(s) & 1 \end{array}\right]}^{T}$ in the single camera coordinate system of the image point ${\left(s,l\right)}_{i}$ on the single virtual detector can be determined as follows:

(4) $$\begin{array}{l} x_{i}(s)=A_{0i}\\ y_{i}(s)=B_{0i}+B_{1i}s \end{array}$$

where i=A,B, $A_{0i}$ and $B_{0i},B_{1i}$ are the interior LOS coefficients of the single virtual detector.

The serial number of virtual detectors A and B can be represented by $S_{A}=0,\ldots ,N_{A}-1$, and $S_{B}=0,\ldots ,N_{B}-1$. $R_{A}^{body}$ and $R_{B}^{body}$ are the installation matrices from the camera A and B coordinate systems to the satellite body coordinate system. As shown in Figure 1c, we can determine the endpoint LOSs for single virtual detectors A and B, $A_{start}:{\left[\begin{array}{ccc} \bar{x_{A0}} & \bar{y_{A0}} & \bar{z_{A0}} \end{array}\right]}^{T}$, $A_{end}:{\left[\begin{array}{ccc} \bar{x_{A1}} & \bar{y_{A1}} & \bar{z_{A1}} \end{array}\right]}^{T}$, $B_{start}:{\left[\begin{array}{ccc} \bar{x_{B0}} & \bar{y_{B0}} & \bar{z_{B0}} \end{array}\right]}^{T}$, $B_{end}:{\left[\begin{array}{ccc} \bar{x_{B1}} & \bar{y_{B1}} & \bar{z_{B1}} \end{array}\right]}^{T}$ as follows:

(5) $$\begin{array}{l} \left[\begin{array}{c} \bar{x_{A0}}\\ \bar{y_{A0}}\\ \bar{z_{A0}} \end{array}\right]=R_{A}^{body}\left[\begin{array}{c} x_{A}(0)\\ y_{A}(0)\\ 1 \end{array}\right],\;\left[\begin{array}{c} \bar{x_{A1}}\\ \bar{y_{A1}}\\ \bar{z_{A1}} \end{array}\right]=R_{A}^{body}\left[\begin{array}{c} x_{A}(N_{A}-1)\\ y_{A}(N_{A}-1)\\ 1 \end{array}\right]\\ \left[\begin{array}{c} \bar{x_{B0}}\\ \bar{y_{B0}}\\ \bar{z_{B0}} \end{array}\right]=R_{B}^{body}\left[\begin{array}{c} x_{B}(0)\\ y_{B}(0)\\ 1 \end{array}\right],\;\left[\begin{array}{c} \bar{x_{B1}}\\ \bar{y_{B1}}\\ \bar{z_{B1}} \end{array}\right]=R_{B}^{body}\left[\begin{array}{c} x_{B}(N_{B}-1)\\ y_{B}(N_{B}-1)\\ 1 \end{array}\right] \end{array}$$

Subsequently, the LOS of $A_{start}$, $A_{end}$, $B_{start}$ and $B_{end}$ can be projected to the plane $Z_{b}$ to obtain their components in the $X_{b}$ and $Y_{b}$ directions, therefore, projected coordinates in the satellite body coordinate system for the four endpoints $\left(x_{A0},y_{A0}\right)$, $\left(x_{A1},y_{A1}\right)$, $\left(x_{B0},y_{B0}\right)$, and $\left(x_{B1},y_{B1}\right)$ can be calculated as follows:

(6) $$\begin{array}{c} x_{A0}=\frac{\bar{x_{A0}}}{\bar{z_{A0}}},y_{A0}=\frac{\bar{y_{A0}}}{\bar{z_{A0}}},\;x_{A1}=\frac{\bar{x_{A1}}}{\bar{z_{A1}}},y_{A1}=\frac{\bar{y_{A1}}}{\bar{z_{A1}}}\\ x_{B0}=\frac{\bar{x_{B0}}}{\bar{z_{B0}}},y_{B0}=\frac{\bar{y_{B0}}}{\bar{z_{B0}}},\;x_{B1}=\frac{\bar{x_{B1}}}{\bar{z_{B1}}},y_{B1}=\frac{\bar{y_{B1}}}{\bar{z_{B1}}} \end{array}$$

The projected BVC coordinates of the two endpoints $\left(x_{BVD_{start}},y_{BVD_{start}}\right)$ and $\left(x_{BVD_{end}},y_{BVD_{end}}\right)$ in the satellite body coordinate system can be calculated as follows:

(7) $$x_{BVD_{start}}=\frac{x_{A0}+x_{B0}}{2},\;y_{BVD_{start}}=y_{A0},\;x_{BVD_{end}}=\frac{x_{A0}+x_{B0}}{2},\;y_{BVD_{end}}=y_{B1}$$

The BVC can be designed with no internal distortion by dividing the interior LOS equally according to the number of virtual detectors, and we can subsequently determine the LOS ${\left[\begin{array}{ccc} x_{V}(s) & y_{V}(s) & 1 \end{array}\right]}^{T}$ of the detector $S\left(s=0,\ldots ,N_{V}\right)$ in the satellite body coordinate system as follows:

(8) $$x_{V}(s)=x_{BVD_{start}},\;y_{V}(s)=y_{BVD_{start}}+\frac{y_{BVD_{end}}-y_{BVD_{start}}}{N_{V}-1}s$$

Then, we can determine the BVC rigorous imaging model as follows:

(9) $${\left[\begin{array}{c} X\\ Y\\ Z \end{array}\right]}_{WGS84}={\left[\begin{array}{c} X_{s}\\ Y_{s}\\ Z_{s} \end{array}\right]}_{WGS84}+\lambda R_{J2000}^{WGS84}R_{body}^{J2000}R_{camera}^{body}\left[\begin{array}{c} x_{v}\left(s\right)\\ y_{v}\left(s\right)\\ 1 \end{array}\right]$$

The advantage of placing the virtual detector in the center line of the double-camera’s detectors along the track direction is to reduce the mosaic and virtualization errors. Based on the determined BVC rigorous imaging model, the corresponding RFM can be determined to generate the complete stitched images.

### 2.3. Image Mosaic Workflow Based on VSD and BVC

**Figure 2:**
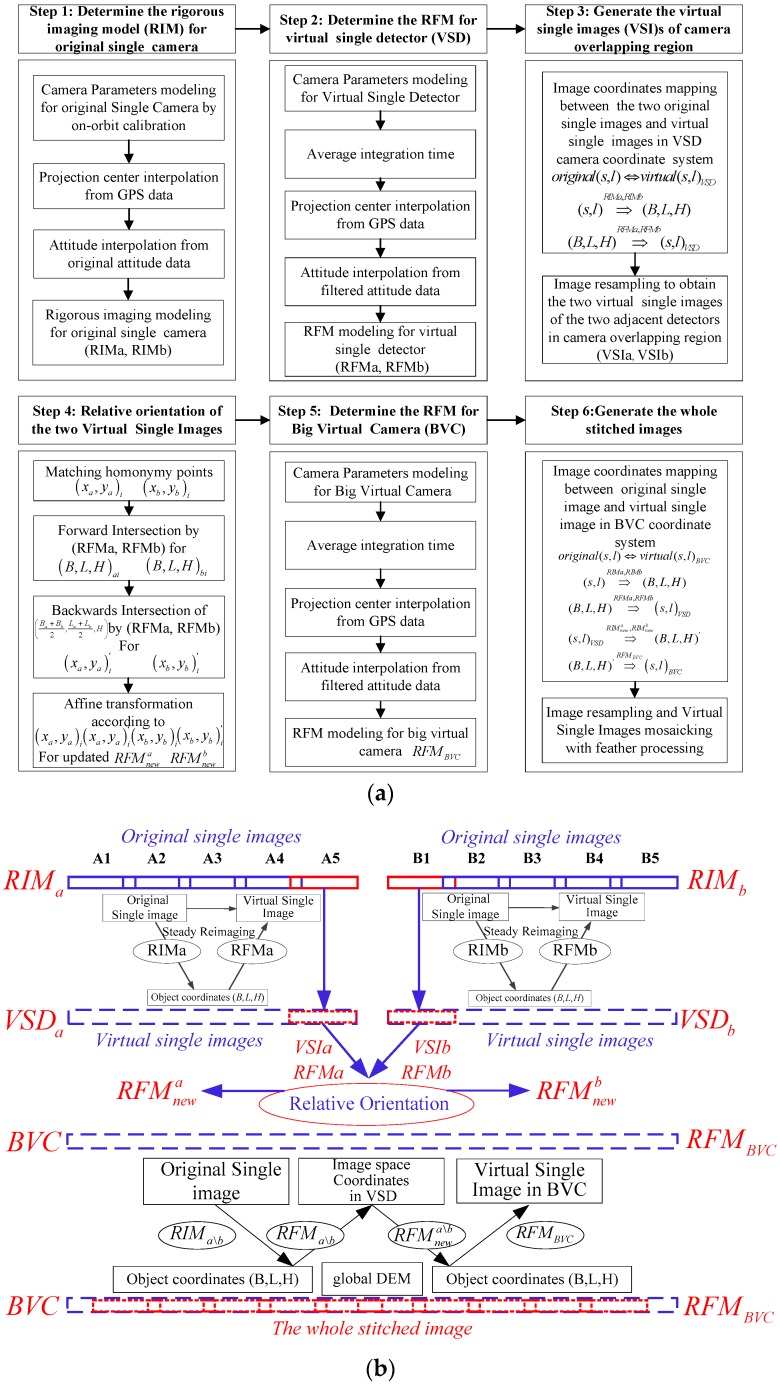
The image mosaic workflow based on the virtual single detector and the big virtual camera: (**a**) Flow diagram; (**b**) Schematic diagram.

One straightforward method to produce the complete stitched image is to generate the stitched image from each single camera first, subsequently, stitch the two images to generate the complete stitched image from the double-cameras. However, it can be time-consuming to resample all the original single images twice. The image mosaic method workflow is based on the virtual single detector and the big virtual camera is designed with six primary steps as shown in Figure 2a.

Step 1: Determine the rigorous imaging model (RIM) for the original single camera. The $RIM_{a}$ and $RIM_{b}$ for the double-cameras can be constructed based on the collinearity condition equation. The high accuracy camera parameters on the satellite is necessary in the proposed mosaicking approach. The camera parameters that include the exterior installation angles and interior LOS parameters can be determined using an on-orbit high accuracy geometric calibration method [20,21,22]. The projection center and attitude of each imaging line can be interpolated from the original GPS and attitude data.

Step 2: Determine the RFM for the virtual single detector (VSD). The VSD shares the same installation angles with RIM and the interior VSD LOS can be designed with no distortion according to the RIM field of view. Based on the new integration time from averaging the original integration times, the interpolated projection center and the smoothed attitude data can be obtained to determine $RFM_{a}$ and $RFM_{b}$ for the left and right VSDs. The $RFM_{a}$ and $RFM_{b}$ are the steady-state reimaging model of $VSD_{a}$ and $VSD_{b}$, and an excellent replacement for $RIM_{a}$ and $RIM_{b}$, which have filtered the observation noise in the auxiliary data through the least square method and, thus, has a more accurate fitting precision [18].

Step 3: Generate the virtual single images (VSI)s of the camera overlap region. Although the consistency of the image positioning accuracy between the adjacent TDI-CCD detectors can be ensured after an on-orbit high accuracy calibration, the consistency of the image positioning accuracy between the adjacent double cameras is hard to maintain due to the inevitable changes of their relative installation relationship caused by the external environmental temperature, which will lead to the dislocation of the camera overlap region. Therefore, to ensure seamless mosaicking solely based on the geometrical information, the relative orientation of the $RFM_{a}$ and $RFM_{b}$ should be determined. The two original single images obtained by the leftmost TDI-CCD detector in the right single camera and the rightmost TDI-CCD detector in the left single camera are projected to their corresponding VSD according to the $RFM_{a}$ and $RFM_{b}$, and their VSIs are generated using image resampling for the following relative orientation. Forward-projection is based on the rigorous imaging model and backward-projection is based on the RFM in steady-state reimaging processing, as shown in Figure 2b.

Step 4: The relative orientation of the two virtual single images (VSIs). Many homonymy points for the two adjacent VSIs can be matched automatically using the SFIT algorithm, and the image space coordinates ${\left(x_{a},y_{a}\right)}_{i}$ are in $VSI_{a}$ and ${\left(x_{b},y_{b}\right)}_{i}$ are in $VSI_{b}$. The corresponding object space coordinates ${\left(B,L,H\right)}_{ai}$ and ${\left(B,L,H\right)}_{bi}$ for ${\left(x_{a},y_{a}\right)}_{i}$ and ${\left(x_{b},y_{b}\right)}_{i}$ can be determined using forward intersection based on $RFM_{a}$ and $RFM_{b}$. Subsequently, the elevation H can be interpolated from the global DEM such as SRTM according to the average object space coordinates ${\left(\frac{B_{a}+B_{b}}{2},\frac{L_{a}+L_{a}}{2}\right)}_{i}$. The corresponding updated image space coordinates ${\left(x_{a},y_{a}\right)}_{i}^{\prime }$ and ${\left(x_{b},y_{b}\right)}_{i}^{\prime }$ in $VSI_{a}$ and $VSI_{b}$ of the same corresponding object space coordinate ${\left(\frac{B_{a}+B_{b}}{2},\frac{L_{a}+L_{a}}{2},H\right)}_{i}$ can be determined using backward intersection based on $RFM_{a}$ and $RFM_{b}$. Finally, the affine transform coefficients of the updated $RFM_{new}^{a}$ and $RFM_{new}^{b}$ can be calculated based on the point pairs ${\left(x_{a},y_{a}\right)}_{i}$ and ${\left(x_{a},y_{a}\right)}_{i}^{\prime }$ in $VSI_{a}$ and ${\left(x_{b},y_{b}\right)}_{i}$ and ${\left(x_{b},y_{b}\right)}_{i}^{\prime }$ in $VSI_{b}$. With the updated $RFM_{new}^{a}$ and $RFM_{new}^{b}$, the consistency of $VSD_{a}$ and $VSD_{b}$ positioning accuracy can be ensured, which is the key factor for generating the complete stitched image from the double-cameras.

Step 5: Determine the RFM for the big virtual camera (BVC). Based on the rigorous imaging model of the single camera, the rigorous imaging model of the big virtual camera can be determined, and combined with the steady-state processed auxiliary data, the high accuracy $RFM_{BVC}$ of the complete stitched image can be produced, which will be applied to the final complete stitched image.

Step 6: Generate the complete stitched images. To generate the virtual single images in the BVC coordinate system, twice forward and backward intersections are performed.

(1) First, for the image point ${\left(s,l\right)}_{original}$ of the original single image, the detector number s determines the interior orientation elements, and imaging line l determines the imaging time so that the exterior orientation elements can be interpolated by time from the attitude and orbit observation. Thus, the corresponding object space coordinate (B,L,H) of image point ${\left(s,l\right)}_{original}$ will be calculated using the forward projection on elevation surface H using the rigorous imaging model. H can be interpolated from the reference elevation surface (such as SRTM DEM).

(2) The image point ${\left(s,l\right)}_{VSD}$ on the corresponding virtual single detector will be calculated using the backward projection from the object space coordinate (B,L,H) using the VSD RFM.

(3) Then, the updated object space coordinate (B,L,H) can be forward intersected from the image point ${\left(s,l\right)}_{VSD}$ based on the $RFM_{new}^{a\backslash b}$ updated using the relative orientation, and H is interpolated from the reference elevation surface.

(4) The final image point ${\left(s,l\right)}_{BVC}$ on the big virtual detector can be backward intersected from the object space coordinate (B,L,H) based on the $RFM_{BVC}$.

(5) Thus, point ${\left(s,l\right)}_{BVC}$ on the big virtual detector and point ${\left(s,l\right)}_{original}$ on the original single image can be correlated. The digital number of ${\left(s,l\right)}_{BVC}$ will be determined from ${\left(s,l\right)}_{original}$ using gray resampling.

(6) Repeat the above steps until every pixel for all the single virtual images on the big virtual detector is complete; then, ten single virtual images can be produced.

(7) Finally, the single virtual images produced will be stitched based on the same initial pixel coordinates for the big virtual detector in the big virtual camera, and feather processing without performing the local correction in the overlapping region can produce the seamless mosaic image.

### 2.4. Error Analysis of the Mosaic Imaging Process

#### 2.4.1. Consistency of the Image Positioning Accuracy

**Figure 3:**
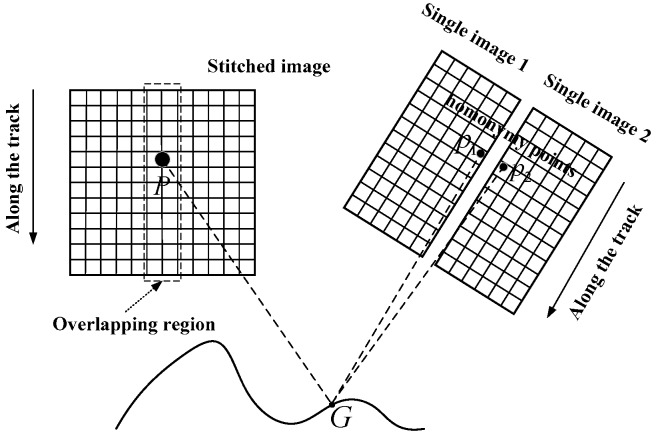
Consistency of the homonymy points positioning accuracy.

Because the geometric imaging model is important in the proposed mosaicking approach, the consistency of the TDI-CCD detectors’ positioning accuracy is the geometric foundation for the mosaic accuracy. As shown in Figure 3, *G* is the object space point of the image space point *P* in the overlapping region of the stitched image. The image space points *P*_1_ and *P*_2_ in the adjacent images are determined using backward-projection from *G* to achieve the conjugate relationship; this means that *P*_1_ and *P*_2_ are homonymy points. In other words, the corresponding object space points *G*_1_ and *G*_2_ of *P*_1_ and *P*_2_ based on their own geometric imaging model should be as close as possible and the distance of the two object space points, which reflects the consistency of the positioning accuracy between the adjacent single images.

The positioning accuracy is determined by the accuracy of the satellite auxiliary data and the camera parameters. Using an on-orbit high accuracy geometric calibration [16,17] for the double-cameras, the camera interior LOS parameters can be continuously verified, while small changes will exist in the installation angles due to the changing thermal environment. Therefore, for the detector overlap region of the adjacent collinear TDI-CCD detector, the adjacent TDI-CCD detector scans the same object line at approximately the same time; then, the same attitude and orbit auxiliary data can be interpolated at the same time. Therefore, the consistency of their positioning accuracy can be achieved. For the camera overlap region of the adjacent cameras, the imaging time of the homonymy points is different due to the inevitable installation error, which causes the interpolated auxiliary data of the same scanning line to be slightly different [19]. Additionally, the unstable relative installation relationship will directly cause the consistency of their positioning accuracy not meet the seamless mosaicking requirements. Therefore, the relative orientation should be determined for the double-cameras.

#### 2.4.2. Influence of the Elevation Error

The difference between the interpolated elevation and the actual elevation that is caused by the limit of the absolute positioning accuracy and the elevation data precision is another key factor for the mosaic accuracy.

**Figure 4:**
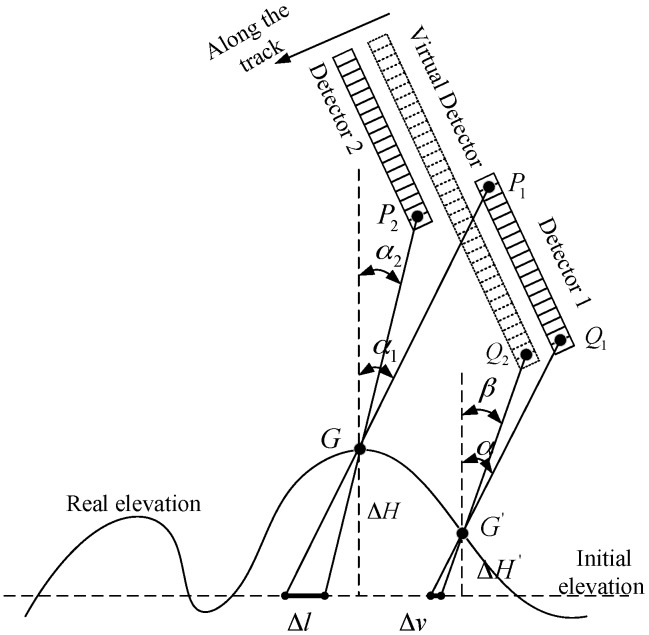
The influence of the elevation error.

As shown in Figure 4, in the overlapping region of the adjacent single image, *G* is the intersection point of the homonymy points *P*_1_ and *P*_2_ for the actual elevation, and Δ*H* is the elevation error. Δ*l* represents the object space projection distance of the mosaic error caused by Δ*H*, as:

(10) $$\Delta l=\Delta H\times \left|\tan\alpha_{1}-\tan\alpha_{2}\right|$$

where the directional angles *α*_1_ and *α*_2_ of the homonymy points *P*_1_ and *P*_2_ are the bias FOV angle of the single CCD detector in the satellite body coordinate system along the track.

For the non-overlapping area, Δ*v* represents the virtualization error caused by the elevation error as follows:

(11) $$\Delta v=\Delta H^{\prime }\times \left|\tan\alpha -\tan\beta \right|$$

where *α* and *β* represent the bias FOV angles of the image homonymy point *Q*_1_ on the original single image and *Q*_2_ on the corresponding single virtual image.

The larger the intersection angle of the homonymy point’s LOS, the greater the influence of the elevation error will be, therefore, to ensure the mosaic and virtualization accuracy, the required interpolated elevation accuracy should be achieved. The elevation error will rarely affect the mosaic accuracy of the detector overlap region due to collinear installation, but has a significant effect on the mosaic accuracy of the camera overlap region. Placing the virtual detector in the center line of the double-camera’s TDI-CCD detectors along the track direction can divide the elevation error equally to all the detectors to ensure the mosaic and virtualization accuracy. After the on-orbit high accuracy calibration and relative orientation, the accuracy of the image positioning and the interpolated elevation from the global DEM can be ensured. Based on the analysis above, the goal is to achieve a seamless mosaic image using image feather processing when stitching the single BVC virtual images directly based on their image coordinate for the same big virtual detector coordinate system.

## 3. Experiments and Discussion

### 3.1. Experimental Data

Four sets of double-panchromatic-images from the GF2 satellite were used in the experiments to verify the reliability of the proposed approach. Table 2 lists the primary information for the images. The four study areas are located in Songshan, Anyang and Dongying, China, and the double-images have the same size. Songshan is a mountainous area with an elevation difference of 1359 m and an average elevation of 431 m, while Anyang and Dongying are flatlands with small elevation differences. Scenes A and B, images of Songshan, were captured in the same orbit on October 27, 2015. The experimental data are the panchromatic images after radiometric-correction, and the same high accuracy calibrated camera parameters and their corresponding auxiliary data. Digital orthophoto maps (DOM) and digital elevation models (DEM) obtained from the WorldView2 satellite was used as reference data for the geometric accuracy evaluation. The DOM resolution is 0.3 m, and the DEM resolution is 2 m. To ensure the appropriate number and distribution of the auto-matched ground control points (GCPs) is used for the geometric accuracy assessment, the selected satellite images have little cloud and water cover. The matching process was performed using the SIFT algorithm, and the matching accuracy was better than 0.3 pixels [23].

Visual and geometric accuracy evaluation of the complete stitched images were performed to comprehensively validate the effectiveness of our approach.

### 3.2. Results and Discussion

#### 3.2.1. Visual Evaluation

**Figure 5:**
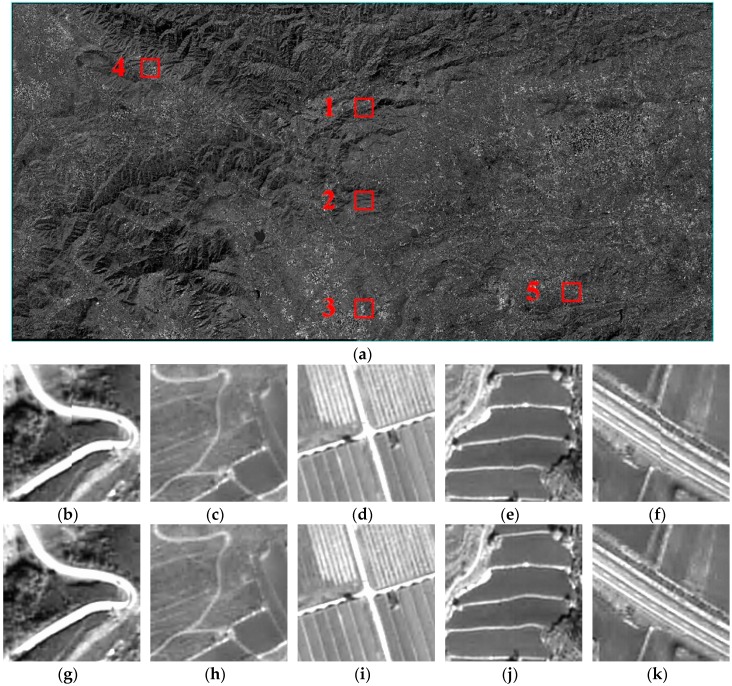
Experimental results of the GF2 satellite imagery: (**a**) The complete stitched image; (**b**) Original overlapping area 1; (**c**) Original overlapping area 2; (**d**) Original overlapping area 3; (**e**) Original overlapping area 4; (**f**) Original overlapping area 5; (**g**) Updated overlapping area 1; (**h**) Updated overlapping area 2; (**i**) Updated overlapping area 3; (**j**) Updated overlapping area 4; (**k**) Updated overlapping area 5.

Based on the high accuracy and stable interior calibrated parameters, seamless mosaicking of the detector overlapping region can be achieved directly based on the geometric relationship. Then feather processing is performed during the local processing, therefore, a significant dislocation of the seamline will occur if there is a significant image positioning deviation for the homonymy points on the adjacent single CCD detector, which can be effectively detected by visual evaluation. Due to the unstable small changes in the relative installation relationship of the double-cameras, relative orientation is required for the seamless mosaicking of the camera overlap region. Figure 5 shows the experimental results for Scene A GF2 satellite imagery. Areas 1, 2 and 3 are camera overlapping regions, while area 4 is the detector overlapping region between detector 2 and 3 in camera A, and area 5 is the detector overlapping region between detector 3 and 4 in camera B. The details of areas 1–5 in Figure 5a are shown in Figure 5b–k. Areas 1, 2, and 4 are in mountainous areas, and areas 3 and 5 are in plain areas.

Areas 4 and 5 could achieve seamless mosaicking when feather processing is performed for the detector overlapping regions. That is due to the high accuracy and stable on-orbit calibrated interior parameters, which are the most important factors for the consistency of the positioning accuracy in the single camera. We can also see that the stitched areas of the camera overlap regions are also seamless and smooth without a local correction after relative orientation with translation transformation. The stitching result will not be affected by the overlapping region terrain, which is benefited from the geometric characteristics we talk about in the following part.

#### 3.2.2. Geometric Accuracy Evaluation

##### RFM Fitting Precision

**Figure 6:**
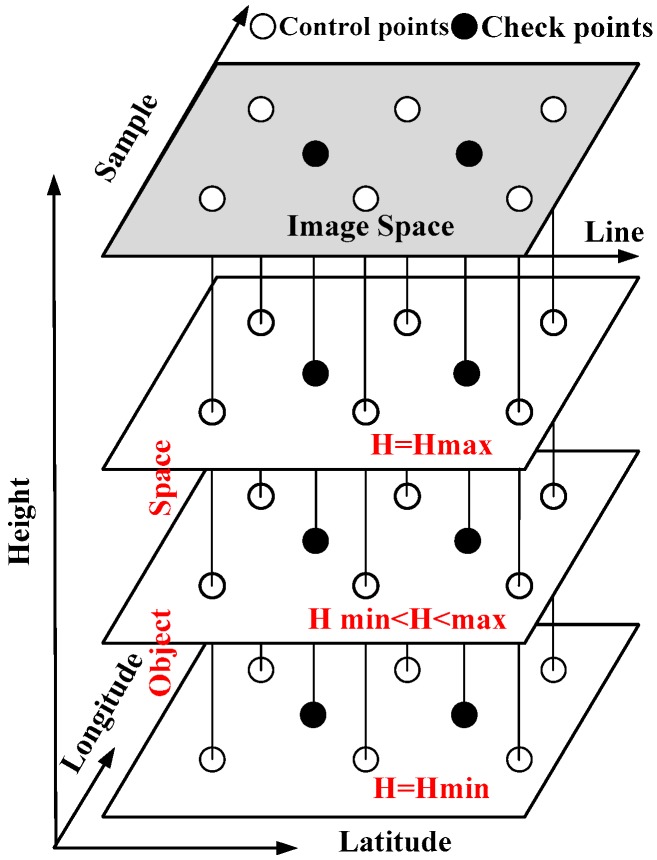
The schematic diagram of the virtual control points and check points.

The RFM fitting precision is one key index of the geometry quality for the complete stitched image, and it can be calculated using check points evenly distributed among the virtual control points when fitting the RFM based on the rigorous imaging model as shown in Figure 6 with the results shown in Table 3.

The maximum and minimum error absolute values for the rational function coefficients (RPCs) fit using the original attitude reaches approximately 0.65 pixels in the sample and 0.93 pixels in the line directions, respectively. However, the RMSE of the RPC fit using the smooth attitude becomes better than 1.00 × 10^−4^ in the sample and line directions, which may be ignored because it fully achieves the accuracy requirement. This is due to the virtual detector of the big virtual camera being designed without internal distortion and external attitude data in the steady-state processing, which are interpolated using the polynomial model that can filter the observation noise using the least square method (LSM). The high RFM accuracy for the complete stitched image obtained using the big virtual camera provides the necessary geometric information for post processing.

##### Mosaic Accuracy

The mosaic accuracy we focus on can be divided into the mosaic accuracy of the detector overlap region and the camera overlap region. Due to the collinear installation of the multi-TDI-CCD detectors, the same scanning line in the detector overlap region is concurrently imaged; then, the auxiliary data are concurrently interpolated by time. Based on the high-accuracy camera interior LOS parameters, the mosaic accuracy of the detector overlap region can stably reach approximately zero pixels. Therefore, feather processing is needed for seamless mosaicking. The mosaic accuracy of the camera overlap region is difficult to ensure using an on-orbit calibration due to the inconsistent relative installation relationship of the double-cameras caused by the changing thermal environment. Therefore, the relative orientation should be performed for the double-images captured by the double-cameras. Theoretically speaking, affine transformation should be performed in the relative orientation considering the rigorous installation relationship. However, the small imaging overlap region of the double-cameras and the complicated imaging terrain features cause the homonymy points auto-matching to be difficult and not completely reliable in most cases. Therefore affine transformation will probably cause a more unreliable geometric accuracy for the double images, especially at the edge of the double-images away from the overlapping region. Considering these problems, translation transformation may be an alternative choice if it can also achieve the ideal mosaic accuracy.

**Figure 7:**
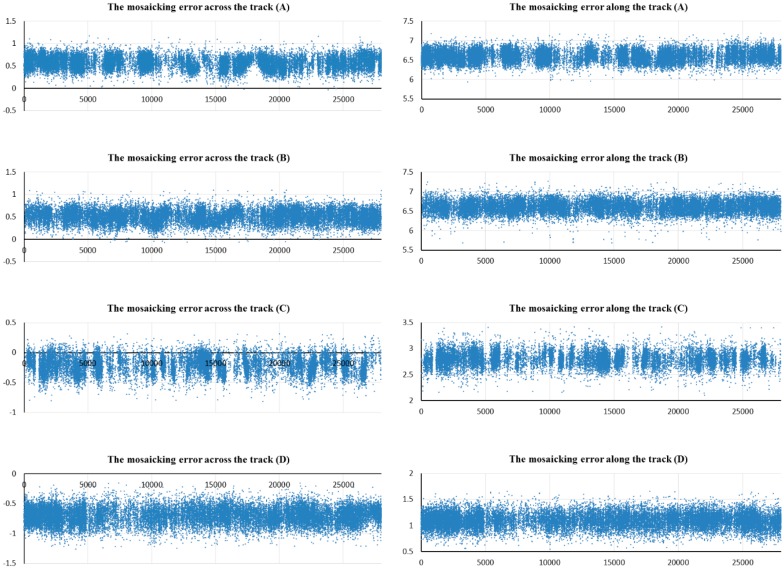
Mosaicking error for the camera overlap region with line number based on original RFM.

Based on the original RFM of virtual single detector, the adjacent single images can be projected to the big virtual detector according to Step 3 in the workflow. Subsequently, the mosaic accuracy can be evaluated using the consistency of the homonymy points in the overlapping regions. More than ten thousand evenly distributed dense homonymy points were matched automatically using the SIFT algorithm. (*s*,*l*)*_left_* is the image coordinate of the matched point in the left single virtual image, and (*s*,*l*)*_right_* is the image coordinate of the matched corresponding homonymy point in the adjacent right single virtual image. Because the homonymy points image coordinates of the left and right single virtual image are in the same big virtual detector plane coordinate system, the homonymy points image coordinates should be the same under ideal conditions. The deviation of the homonymy points’ image coordinates will directly affect and reflect the mosaic accuracy. The mosaic errors (pixels) relationship with the line number are shown in Figure 7. Significant mosaic errors occur across and along the track direction. The mosaic error fluctuations for the four scenes in the camera overlap region are small, which verifies the consistency of the auxiliary data measurements accuracy in the small imaging time interval of the double-cameras caused by the small displacement installation. The mosaic errors of scenes A and B in the same orbit are approximately the same, and they have significant differences compared to scenes C and D, which were imaged on a different day. Considering the use of high accuracy elevation information, such as GDEM2 for generating the virtual image, the inconsistent mosaic errors are probably caused by the change of the relative installation relationship for the double-cameras. Based on the densely distributed homonymy points in the camera overlap region, translation and affine transformations for the updated RFMs of the single camera are performed using the method in Step 4 of the workflow. The affine model based on RFM was used as the exterior orientation model [24,25]. Based on the updated RPMs, the mosaic accuracy statistics are re-calculated, and the results are shown in Table 4.

(12) $$\begin{array}{l} x+a_{0}+a_{1}x+a_{2}y=RFM_{x}(lat,\;lon,\;h)\\ y+b_{0}+b_{1}x+b_{2}y=RFM_{y}(lat,\;lon,\;h) \end{array}$$

In Table 4, the mosaic error across and along the track directions after translation and affine transformations can be significantly reduced. There is no significant advantage for using the affine transformation compared to the translation transformation, thus, we can speculate that the relative installation change is primarily caused by the changing pitch and roll angles, while the yaw angle change is very small and can be ignored. In addition, a simple calculation shows that the plane mosaic accuracy is better than one pixel, and feather processing is also suitable for the seamless mosaicking of the camera overlap region after the relative orientation is determined.

##### Positioning Accuracy

Based on the updated RFMs obtained in Step 4, the complete stitched image can be generated using Step 5 and Step 6 in the workflow. To evaluate the performance of the proposed image mosaicking approach for maintaining the image position accuracy, the absolute and relative positioning accuracy for the complete stitched image and the single camera image from the single camera with the original RFM was analyzed. We extracted the corresponding DOM and DEM, and 64 GCPs for the complete stitched image and 32 GCPs for the single camera image were matched to evaluate the positioning accuracy. The absolute positioning accuracy is the image positioning accuracy without GCPs, and all GCPs were performed as check points to evaluate the absolute positioning accuracy. The internal relative positioning accuracy can be evaluated using the positioning accuracy with a few GCPs. An affine transformation of the image was applied based on 4 GCPs in the 4 corners of the image to eliminate the exterior systematic offset of the positioning, and the internal relative accuracy statistics were calculated based on the other GCPs. The RFM affine model was also used as the exterior orientation model [24,25].

The geometric accuracy evaluation results of the stitched images are listed in Table 5. The absolute positioning accuracy of the complete virtual image was approximately equal to the absolute positioning accuracy of the two single camera images due to the big virtual detector being placed in the center line of the double-camera detectors along the track direction. Additionally, the relative positioning accuracy of the complete virtual images was also approximately equal to one of the two single camera images. Though approximately 0.1 pixels for the internal relative accuracy loss occur after mosaicking, it still performed well in maintaining the original geometric accuracy. In addition, the internal relative accuracy achieved by the affine transformation is slightly better than the translation transformation, which benefits from the well matched dense homonymy points in the camera overlap region. However, in most cases, due to cloud coverage and complicated terrain, dense evenly distributed homonymy points are difficult to obtain for the coefficient calculations for the high accuracy affine transformation, which will lead to instability of the image edge away from the camera overlap region. However, the translation transformation is a much simpler method, and it can be achieved using a few homonymy points and has better practicability, therefore, it is the optimal method in the relative orientation. Thus, we conclude that the proposed mosaicking approach has no considerable negative effects on the absolute and relative positioning accuracy.

## 4. Conclusions and Future Work

In this paper, we have proposed an automatic image mosaicking approach for the double-camera system on the optical remote sensing satellite GF2, and subtly used the concept of the big virtual camera to obtain the stitched image and the corresponding high accuracy RFM for post concurrent processing. The following conclusion may be drawn from our results:

1) Based on the rigorous imaging model for the single camera, the rigorous imaging model for the big virtual camera was established, which would exactly apply to the complete stitched image. Additionally, the image mosaic workflow based on the virtual single detector and the big virtual camera were presented in detail.
2) High accuracy camera parameters on the satellite are necessary in the proposed mosaicking approach. Therefore, high accuracy on-orbit geometric calibration is the precondition to guarantee the effectiveness of our approach.
3) Benefiting from the platform stability and the small relative installation error, the mosaic error of the camera overlap region could be controlled in one pixel after an on-orbit high accuracy geometric calibration and the relative orientation, otherwise, a local correction may be required to achieve seamless mosaicking.
4) Cloud coverage and complex terrain in the camera overlap region may influence the homonymy point matching. Although we verified the ability of the translation transformation in the relative orientation to reduce the dependence on the homonymy point quantity and quality, it still cannot be applied to all situations.
5) The relative installation instability of the double-cameras and the high-frequency platform vibration are key factors in achieving the seamless mosaic from the double-cameras, therefore, if they can be securely attached in the future satellite platform, a simpler workflow without relative orientation can achieve a more ideal mosaic result.

## Figures and Tables

**Table 1:** Information for the single camera on the GF2 satellite.

Information	Multispectral Sensor	Panchromatic Sensor
Spectral range	B1: 450~520 nm	Pan: 450~900 nm
	B2: 520~590 nm	
	B3: 630~690 nm	
	B4: 770~890 nm	
Pixel size	40 µm	10 µm
TDI-CCD number of each band	1536 × 5	6144 × 5
Overlapping TDI-CCD number	95 × 4	380 × 4
Ground sample distance	3.24 m	0.81 m
Focal length	7785 mm	
Field angle	2.1°	
Quantization bits	10	

**Table 2:** Experimental data information.

Study Area	Images	Imaging Date	Satellite Attitude Roll/Pitch/Yaw (Degree)			$H_{aver}/H_{diff}$ *	Image Size
							Camera A/B
Songshan	Scene A	27 October 2015	12.99870	0.00111	2.99763	431/1359	29,200 × 27,620
Songshan	Scene B	27 October 2015	12.99870	0.00091	3.00424	431/1359	29,200 × 27,620
Anyang	Scene C	20 October 2015	−7.00335	−0.00039	3.04978	39/98	29,200 × 27,620
Dongying	Scene D	16 December 2016	−4.00286	−0.00014	3.00613	4/23	29,200 × 27,620

* $H_{aver}$ denotes the average height and $H_{diff}$ denotes the height difference.

**Table 3:** Statistical results comparison of the RPCs fit using a smooth attitude.

Scene	Statistic (Pixels)	Oscillating Attitude		Smooth Attitude	
		Sample	Line	Sample	Line
A	Mean	−2.01 × 10^−4^	−3.92 × 10^−5^	−1.05 × 10^−6^	−1.78 × 10^−7^
	RMSE	0.13	0.43	3.37 × 10^−5^	3.03 × 10^−5^
	Maximum	0.65	0.79	8.99 × 10^−5^	6.78 × 10^−5^
	Minimum	−0.54	-0.88	−9.01 × 10^−5^	−6.80 × 10^−5^
B	Mean	−1.41 × 10^−4^	−4.57 × 10^−5^	−1.82 × 10^−6^	−2.28 × 10^−7^
	RMSE	0.11	0.40	2.96 × 10^−5^	3.19 × 10^−5^
	Maximum	0.53	0.81	7.17 × 10^−5^	8.90 × 10^−5^
	Minimum	−0.28	−0.84	−7.12 × 10^−5^	−8.84 × 10^−5^
C	Mean	4.47 × 10^−4^	−3.40 × 10^−6^	−3.44 × 10^−6^	−1.78 × 10^−7^
	RMSE	0.16	0.32	3.61 × 10^−5^	2.48 × 10^−5^
	Maximum	0.57	0.83	9.27 × 10^−5^	7.78 × 10^−5^
	Minimum	−0.42	−0.93	−9.22 × 10^−5^	−7.80 × 10^−5^
D	Mean	−2.63 × 10^−6^	−4.20 × 10^−6^	−2.22 × 10^−6^	−1.50 × 10^−7^
	RMSE	0.23	0.32	3.26 × 10^−5^	5.12 × 10^−6^
	Maximum	0.57	0.74	8.10 × 10^−5^	1.00 × 10^−5^
	Minimum	−0.57	−0.88	−8.00 × 10^−5^	−9.00 × 10^−6^

**Table 4:** Mosaic accuracy of the camera overlap region using different transformations.

Transformation	Error	Scene A		Scene B		Scene C		Scene D	
		Sample	Line	Sample	Line	Sample	Line	Sample	Line
Translation	Maximum	0.59	0.58	0.59	0.65	0.52	0.60	−0.55	0.56
	Minimum	−0.60	−0.66	−0.58	−0.69	−0.61	−0.69	−0.55	−0.55
	RMSE	0.13	0.14	0.13	0.14	0.14	0.13	0.14	0.13
Affine	Maximum	0.59	0.59	0.60	0.64	0.53	0.60	−0.54	0.56
	Minimum	−0.61	−0.63	−0.59	−0.69	−0.60	−0.68	−0.55	−0.55
	RMSE	0.13	0.14	0.13	0.14	0.14	0.13	0.14	0.13

**Table 5:** Geometric accuracy evaluation of the complete stitched images.

		Absolute Positioning Accuracy		Relative Positioning Accuracy	
Scene	Sensor	X/Pixel	Y/Pixel	X/Pixel	Y/Pixel
A	Camera A	−14.81	−26.84	0.90	0.89
	Camera B	−14.84	−20.67	0.95	0.92
	Virtual Camera by TT	−14.80	−28.26	1.07	1.12
	Virtual Camera by AT	−13.10	−21.41	1.01	1.05
B	Camera A	−13.89	−29.56	0.92	0.89
	Camera B	−14.24	−29.21	0.94	0.91
	Virtual Camera by TT	−14.15	−29.23	1.05	1.08
	Virtual Camera by AT	−13.50	−20.86	1.04	1.02
C	Camera A	9.62	12.92	0.89	0.85
	Camera B	7.88	11.20	0.92	0.86
	Virtual Camera by TT	9.13	12.33	1.12	0.98
	Virtual Camera by AT	9.93	11.18	1.00	0.96
D	Camera A	−19.19	−23.16	0.91	0.90
	Camera B	−18.51	−29.24	0.92	0.92
	Virtual Camera by TT	−19.23	−27.27	1.02	1.08
	Virtual Camera by AT	−18.10	−28.35	0.98	0.99
